# Small Food Stores and Availability of Nutritious Foods: A Comparison of Database and In-Store Measures, Northern California, 2009

**DOI:** 10.5888/pcd9.120023

**Published:** 2012-07-12

**Authors:** Ellen Kersten, Barbara Laraia, Maggi Kelly, Nancy Adler, Irene H. Yen

**Affiliations:** Author Affiliations: Barbara Laraia, Nancy Adler, Irene H. Yen, University of California, San Francisco, California; Maggi Kelly, University of California, Berkeley, California.

## Abstract

**Introduction:**

Small food stores are prevalent in urban neighborhoods, but the availability of nutritious food at such stores is not well known. The objective of this study was to determine whether data from 3 sources would yield a single, homogenous, healthful food store category that can be used to accurately characterize community nutrition environments for public health research.

**Methods:**

We conducted in-store surveys in 2009 on store type and the availability of nutritious food in a sample of nonchain food stores (n = 102) in 6 predominantly urban counties in Northern California (Alameda, Contra Costa, Marin, Sacramento, San Francisco, and Santa Clara). We compared survey results with commercial database information and neighborhood sociodemographic data by using independent sample *t* tests and classification and regression trees.

**Results:**

Sampled small food stores yielded a heterogeneous group of stores in terms of store type and nutritious food options. Most stores were identified as convenience (54%) or specialty stores (22%); others were small grocery stores (19%) and large grocery stores (5%). Convenience and specialty stores were smaller and carried fewer nutritious and fresh food items. The availability of nutritious food and produce was better in stores in neighborhoods that had a higher percentage of white residents and a lower population density but did not differ significantly by neighborhood income.

**Conclusion:**

Commercial databases alone may not adequately categorize small food stores and the availability of nutritious foods. Alternative measures are needed to more accurately inform research and policies that seek to address disparities in diet-related health conditions.

## Introduction

One aspect of neighborhood context that has received attention from public health researchers and advocates in recent years is the availability of food outlets and nutritious food, commonly referred to as the community nutrition environment (1). Given the strong relationship between diet and health, and the limited availability of sources of nutritious food in many low-income and racial/ethnic minority neighborhoods, community nutrition environments may contribute to disparities in diet-related health conditions, such as obesity, diabetes, and cardiovascular disease (2,3).

To evaluate community nutrition environments, researchers frequently use food store location and classification data from secondary data sources, such as proprietary commercial databases or business listings from public agencies (4). Supermarkets and large chain grocery stores tend to offer a variety of nutritious foods, and access to such stores is related to improved diet and reduced risk for obesity (2,3). However, the classification of small, independently owned (nonchain) food stores remains a challenge. Small, independent food stores have been either ignored (5,6) or distinguished from supermarkets and convenience stores according to the number of cash registers (7), industry codes (8), store name (9,10), number of employees (11,12), or annual sales volume (13). More recent approaches use combinations of characteristics included in commercial databases to categorize independent food stores as either "healthy" or "unhealthy" (14-16).

Small, independent food stores comprise most food retail locations in urban neighborhoods; proper categorization of such stores is important for studies on community nutrition environments. The primary objective of this study was to examine the categorization of small food stores and determine whether data from 3 sources would yield a single homogenous healthful food store category. We hypothesized that most small food stores (defined as generating less than $1 million in annual sales) selected from a single industry category for grocery stores would represent a homogenous group of healthful food stores (ie, offer nutritious and fresh food items). Secondary objectives were to examine the availability of nutritious foods in small food stores across neighborhood sociodemographic contexts and test for inaccuracies in commercial database variables that could bias or misrepresent measures of nutritious food availability.

**Podcast: Interview with Author Ellen Kersten**

Ellen Kersten, a University of California, Berkeley PhD candidate and this year’s winner of PCD’s 2012 student research contest, investigates the availability of nutritious foods in small food stores in six predominantly urban counties in Northern California. PCD interviewed Kersten about her research and asked her what she has planned after graduation. Listen now.

## Methods

### Study design

We used stratified random sampling to select stores from a commercial database to survey. We used in-store surveys to assess store type and the availability of fresh and nutritious food items at selected stores and compared these measures with neighborhood-level sociodemographic characteristics and commercial database attributes. Institutional review board approval was not required for this study because no human participants were involved.

### Study sample

We selected a 6-county study area in the Sacramento and San Francisco Bay Area (Alameda, Contra Costa, Marin, Sacramento, San Francisco, and Santa Clara counties) because 2 authors (B.L. and I.H.Y.) are working on 2 studies in this area. All 6 counties have predominantly urban populations; more than 90% of both food stores and households are located in the urban areas in each county. We identified all small grocery stores in the study area by using 2008 data from InfoUSA (www.infousa.com), a provider of data on commercial establishments, through an Esri Business Analyst extension (Esri, Redlands, California). The InfoUSA database includes attributes for each business location, including industry code (as reported by each business using the North American Industry Classification System [NAICS]), annual sales volume, number of employees, franchise status, and size (categorical square footage). Using the NAICS industry code for "supermarkets and other grocery (except convenience) stores" (445110), we identified 2,400 stores. We excluded stores that had an NAICS code for convenience stores (445120) because they comprise a much smaller portion of the retail food environment (n = 522) than grocery stores do in the study area, and more than half of the convenience stores are chains (eg, 7-Eleven, Circle K) that tend to have a limited availability of nutritious food. Of the grocery stores identified, 1,604 (67%) had an annual sales volume of less than $1 million, which we used to define "small." We used this threshold because it is the lowest value used in previous studies to differentiate between "healthy" and "unhealthy" small food stores in California (14,16). After also excluding stores designated as headquarters or franchises, we had 1,582 small, nonchain food stores in our sample. All of these stores were in the same size category (1–2,499 sq ft) and had fewer than 5 employees. To ensure sampling across the number of employees that has been used to differentiate stores in previous studies (14), we divided the sample into 2 groups: stores that had 2 or fewer employees (n = 1,289 [81%]) and stores with 3 or 4 employees (n = 293 [19%]). We stratified each of the 2 groups by county and by quartile of neighborhood deprivation (17) and randomly selected 5% of the stores from each stratum. Each 5% sample was rounded up to the next whole number, resulting in an initial sample of 102 stores, or 6% of small grocery stores in the study area. Compared with the other small, independent stores in the study area, the stores in this sample had a higher mean annual sales volume ($579,000 vs $642,000) and more employees (mean employee count of 1.5 for all stores vs mean of 1.7 for stores in our sample). Of the 102 stores, we could not survey 15; we could not find 4 stores, 3 were out of business, and 8 were not food stores. No store managers declined to have their stores surveyed. Our final sample included 87 stores.

### In-store survey

We designed a 2-page, 39-question survey to assess each store (Appendix). The survey was adapted from the CX^3^ Food Availability and Marketing Survey created and validated by the California Department of Public Health (18). We conducted surveys from May through early September 2009. In each store, surveyors introduced themselves to store managers, described the survey, and provided a letter, including author contact information, about the study. The survey included questions in 7 main categories: store name and location; exterior characteristics; estimated area in square feet; availability and variety of fresh fruit, vegetables, and raw meat/seafood (coded from 1 to 4, with 4 being the most variety); quality of fresh fruit and vegetables (coded from 1 to 4, with 4 being the highest quality); and presence of 17 nutritious food items (eg, canned and frozen fruits and vegetables, low-fat milk, high-fiber cereal) (coded 1 for presence or 0 for absence). We created 4 categories of store type as the dependent variable for analyses:

- **Large grocery**: a large store that sells food and other items, including canned and frozen foods, fresh fruits and vegetables, and fresh (raw) and prepared meat, fish, and poultry.
- **Small grocery**: usually an independent store that may sell food including canned and frozen foods, fresh fruits and vegetables, and fresh (raw) and prepared meat, fish, and poultry as well as convenience items and alcohol.
- **Convenience**: a store that sells convenience items only, including bread, milk, soda, and snacks and may sell alcohol and gasoline. These stores do not sell fresh (raw) meat.
- **Specialty**: Liquor store, bakery, donut shop, meat or fish markets (predominantly selling fresh/raw meat), or other specialty stores.

The inventory of 17 food items was summed to create a "nutritious food score" (possible range of 0–17), and a "fresh score" was created by summing the coded values for the availability of fresh fruit, vegetables, and raw meat/seafood (possible range of 3–12, with 3 indicating no fresh food and 12 indicating a variety of fresh foods).

### Neighborhood sociodemographic context

We used 2000 US Census data at the tract level (19) to characterize the neighborhood sociodemographic context for each store. A neighborhood deprivation index was created as a continuous variable according to previous methods (17) that used principal component analysis of 8 derived census variables (percentage of people who have an income below poverty level, percentage of female-headed households that have dependents, percentage of households that have an annual income of less than $30,000, percentage of households that have public assistance income, percentage of people aged 16 or older in the civilian labor force who are unemployed, percentage of men in management, percentage of all people aged 25 or older who did not graduate from high school, and percentage of households with more than 1 person per room). The resulting scores ranged from –3.3 to 14.8; the mean (standard deviation [SD]) score was 0 (2.2). The more positive the score, the more deprived the census tract. We divided the index into quartiles for sampling purposes.

We created continuous variables for race/ethnicity according to the percentage of white, Hispanic, black, and Asian populations. We characterized each neighborhood according to population density (total population divided by area in square miles), percentage of children (population aged younger than 18 divided by total population) and elderly (total population 65 or older divided by total population), and neighborhood stability (percentage of population that lived at the same location in 1995 and 2000).

### Statistical analyses

We used independent sample *t* tests to compare the mean differences for fresh food availability among store types and to evaluate the differences between neighborhood sociodemographic context and store type. We used Stata/SE 9.0 (StataCorp, College Station, Texas) for analyses. We conducted classification and regression tree (CART) analysis by using variables from the in-store surveys, the InfoUSA database, and census information to identify store attributes and neighborhood characteristics that most parsimoniously identified store type. This method can handle multiple outcome groups and dichotomous, ordinal, categorical, and continuous explanatory variables, which makes it an ideal method in this analysis, where various attributes are associated with each food store. CART builds a "tree" for classifying the data by finding "nodes," or values of the explanatory variables that significantly differentiate 1 or more outcome groups (20). For the CART analysis, large and small grocery stores were combined into 1 outcome group because these 2 store types represented the same outcome of interest, healthful food availability. Convenience and specialty stores remained separate groups because of their more varied and distinct survey results, for a total of 3 outcome groups. The CART analysis was conducted using the rpart library (21) in the R Statistical Environment 10.1.1 (R Development Core Team, Vienna, Austria).

## Results

### Types of food stores

**Figure 1:**
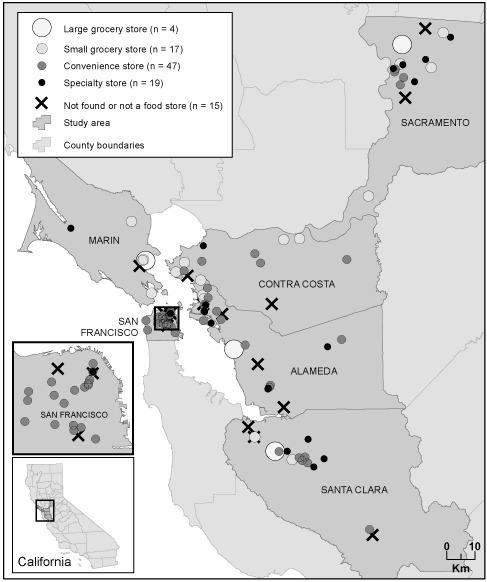
Spatial distribution of food stores surveyed, by store type and county, San Francisco Bay Area and Sacramento, California, 2009.

The 87 stores surveyed were categorized as 4 large grocery stores, 17 small grocery stores, 47 convenience stores, and 19 specialty stores (Figure 1). Of the 19 specialty stores, 12 were ethnic food stores, 3 were liquor stores that sold some microwavable food items, 2 sold only meat and produce, 1 was a delicatessen, and 1 was a wine and cheese store that sold some produce items.

**Table:** 

Store type	County						

	Alameda	Contra Costa	Marin	Sacramento	San Francisco	Santa Clara	Total
**Large grocery store**	1	0	1	1	0	1	4
**Small grocery store**	2	5	4	4	0	2	17
**Convenience store**	11	5	0	4	21	6	47
**Specialty store**	7	1	1	5	1	4	19
**Not found or not a food store**	4	2	1	2	3	3	15
**Total**	25	13	7	16	25	16	102

### Availability of nutritious food

Of the 87 surveyed stores, 53 (61%) sold at least a limited variety (1–3types) of both fresh fruit and vegetables, and 20 stores (23%) sold no fruits or vegetables. Of the 60 (69%) stores that sold at least some fruit, more than half (n = 35) had high- or fair-quality fruit (all good or more good than poor quality). Of the 59 (68%) stores that sold at least some vegetables, more than two-thirds (n = 40) had high- or fair-quality vegetables. Fifty stores (57%) carried more than half of the surveyed items (nutritious food score >8), and 35 (40%) had more than 3 types of fresh fruits, vegetables, and raw meat/seafood (fresh score >6); 26 stores (30%) had both a nutritious food score of 8 or more and a fresh score of 6 or more. Twenty-eight stores (32%) had fewer than half of the surveyed nutritious food items (nutritious food score ≥8) and sold no or a limited variety of fresh foods (fresh score ≥6).

Nutritious and fresh food availability varied by store type (Table 1). All 4 large grocery stores had a variety of nutritious food items and good-quality produce and meat products. The small grocery stores had a greater number of nutritious food items and fresh fruit and vegetables than convenience or specialty stores. Of the stores that carried some fruits or vegetables, larger grocery stores had better-quality vegetables than small grocery stores and better-quality fruits and vegetables than convenience stores. Specialty stores that had some fruit had better-quality fruit than convenience stores.

**Table 1:** Store Characteristics by Store (N = 87) Type, San Francisco Bay Area and Sacramento, California, 2009

Characteristic	Mean (SD) [Range]				
	Large Grocery (n = 4)	Small Grocery (n = 17)	Convenience (n = 47)	Specialty (n = 19)	Total (N = 87)
Nutritious food score (scored from 0–17)	14.8^a,b^ (2.2) [12–17]	11.5^a,b^ (3.1) [4–17]	9.2^b^ (2.7) [4–14]	4.4 (3.6) [0–14]	8.9 (4.0) [0–17]
Fresh food score (scored from 3–12)	12.0^a, b^ (0.0) [12]	9.6^a,b^ (2.2) [5–12]	5.0 (1.9) [3–10]	6.1 (3.0) [3–12]	6.4 (3.1) [3–12]
Fresh fruit variety score (scored from 1–4)	4.0^a,b^ (0.0) [4]	3.2^a,b^ (0.9) [2–4]	2.0 (1.0) [1–4]	1.9 (1.2) [1–4]	2.3 (1.2) [1–4]
Fresh vegetable variety score (scored from 1–4)	4.0^a,b^ (0.0) [4]	3.8^a,b^ (0.7) [2–4]	1.9 (0.9) [1–4]	2.0 (1.2) [1–4]	2.4 (1.2) [1–4]
Meat/seafood variety score (scored from 1–4)	4.0^a,b,c^ (0) [4]	2.6^b^ (1.1) [1–4]	1.1 (0.3) [1–3]	2.1^a^ (1.2) [1–4]	1.7 (1.1) [1–4]
Fresh fruit quality score^d^ (scored from 1–4)	3.3^a^ (0.5) [3–4]	2.8 (0.7) [2–4]	2.4 (0.6) [1–4]	3.0^a^ (0.8) [2–4]	2.7 (0.7) [1–4]
Fresh vegetable quality score^d^ (scored from 1–4)	3.5^a,c^ (0.6) [3–4]	2.9 (0.5) [2–4]	2.6 (0.6) [2–4]	2.4 (1.0) [2–3]	2.7 (0.6) [2–4]
Store area, sq ft	2,745^a,b,c^ (1,819) [1,134–5,248]	381^a,b^ (180) [112–792]	164 (211) [33–1,430]	155 (174) [30–693]	276 (627) [30–5,248]
No. of employees^e^	1.0 (2.0) [0–4]	1.6 (1.2) [0–3]	1.9 (1.1) [0–4]	1.8 (1.4) [0–4]	1.8 (1.2) [0–4]
Annual sales volume,^e^ $100,000	865^a^ (247) [494–988]	688 (243) [247–988]	578 (248) [247–988]	663 (297) [247–988]	627 (263) [247–988]

^a^ Value significantly higher than value for convenience stores (*P* < .05).

^b^ Value significantly higher than value for specialty stores (*P* < .05).

^c^ Value significantly higher than value for small grocery stores (*P* < .05).

^d^ Only stores that sold at least 1 type of fruit/vegetable were included in these comparisons.

^e^ Values reported from InfoUSA database. Values in all other rows are from in-store surveys.

### Other store attributes

According to in-store surveys, large grocery stores had the greatest estimated square footage, and small grocery stores were larger than convenience and specialty stores. According to InfoUSA, the mean number of employees did not differ significantly by store type; large grocery stores had a significantly larger sales volume than convenience stores (Table 1).

### Neighborhood sociodemographic differences by store type

Neighborhood deprivation did not differ by store type (Table 2). However, neighborhoods that had small grocery stores were on average 63% white; neighborhoods that had convenience stores were on average 49% white (*t* = 2.32, *P* = .02). Neighborhoods that had small grocery stores were less densely populated than neighborhoods that had convenience stores (*t* = –2.92, *P* = .005). Neighborhoods that had specialty stores had a larger average percentage of Asians (36%) than neighborhoods that had small grocery (7% Asian; *t* = –3.80, *P* < .001) or convenience stores (22% Asian; *t =* –2.32, *P* = .02). No other sociodemographic measure differed by store type.

**Table 2:** Census Tract Characteristics (Mean [SD]) by Store (N = 87) Type, San Francisco Bay Area and Sacramento, California, 2000

	Large Grocery (n = 4)	Small Grocery (n = 17)	Convenience (n = 47)	Specialty (n = 19)
**Neighborhood deprivation index^a^**	0.73 (2.4)	0.21 (2.1)	0.77 (2.4)	0.67 (2.3)
**Population density, no. of people per square mile**	7,631 (1,249)	7,107 (5,217)	26,340^b^ (26,896)	14,323 (22,799)
**Median annual household income, $**	53,835 (18,432)	51,100 (17,839)	46,812 (18,380)	48,810 (26,167)
**Race/ethnicity, %**				
White	50 (8.6)	63^c^ (23.2)	49 (21.0)	45 (30.7)
Black	7 (9.3)	12 (18.0)	12 (18.3)	7 (11.1)
Hispanic	31 (26.3)	24 (21.1)	21 (19.3)	15 (10.1)
Asian	20 (10.5)	7 (5.0)	22^b^ (18.3)	36^b,c^ (30.8)
**Age group, %**				
Children	27 (7.0)	21 (8.3)	19 (10.0)	21 (7.4)
Elderly	11 (7.4)	12 (7.4)	11 (5.5)	16 (11.6)
**Neighborhood stability**				
People who lived in same tract in 1995 and 2000, %	92 (2.1)	94 (2.5)	95 (2.8)	94 (1.9)

^a^ The more positive the score, the more deprived the census tract. The values are created from principal component analysis of 8 derived census variables: percentage of people who have an income below poverty level, percentage of female-headed households that have dependents, percentage of households that have an annual income of less than $30,000, percentage of households that have public assistance income, percentage of people aged 16 or older in the civilian labor force who are unemployed, percentage of men in management, percentage of all people aged 25 or older who did not graduate from high school, and percentage of households with more than 1 person per room (17). Scores for census tracts in study area ranged from –3.3 to 14.8.

^b^ Value higher than value for small grocery stores (*P* < .05).

^c^ Value higher than value for convenience stores (*P* < .05).

### Classification of stores by data source

**Figure 2:**
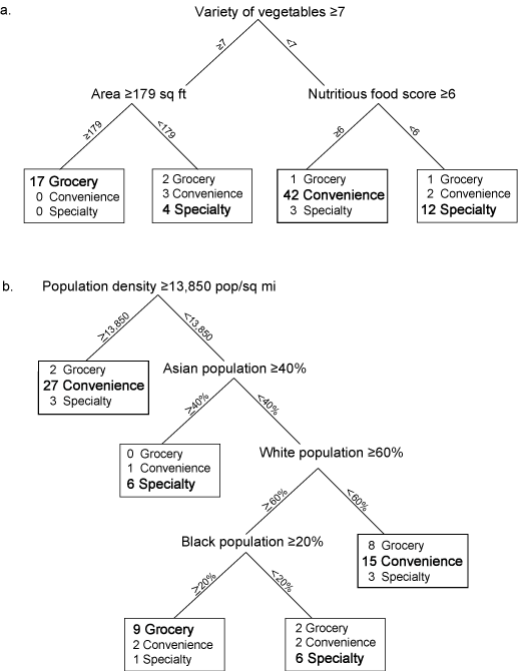
Classification and regression tree results based on a) in-store survey and b) sociodemographic variables.

When we used in-store survey information, we classified 86% of the stores correctly (Figure 2a). The most distinguishing variables were variety of vegetables, estimated store square footage, and nutritious food score. When we used census information, we classified 72% of the stores correctly; neighborhood population density, percentage Asian population, percentage white population, and percentage black population were the most distinguishing variables (Figure 2b). A CART analysis of the InfoUSA values for the number of employees and annual sales volume could not be completed because none of the database variables adequately distinguished store type.

**Table:** 

Distinguishing criteria	Store type		

	Grocery	Convenience	Specialty
**In-store survey (a)**			
**Variety of vegetables ≥7 and area ≥179 sq ft**	17	0	0
**Variety of vegetables ≥7 and area <179 sq ft**	2	3	4
**Variety of vegetables <7 and nutritious food score ≥6**	1	42	3
**Variety of vegetables <7 and nutritious food score <6**	1	2	12
**Sociodemographic variables (b)**			
**Population density ≥13,850 per sq mi**	2	27	3
**Population density <13,850 per sq mi and Asian population ≥40%**	0	1	6
**Population density <13,850 per sq mi, Asian population <40%, and white population <60%**	8	15	3
**Population density <13,850 per sq mi, Asian population <40%, white population ≥60%, and black population ≥20%**	9	2	1
**Population density <13,850 per sq mi, Asian population <40, white population ≥60%, and black population <20%**	2	2	6

## Discussion

A stratified random selection of small, independent food stores drawn from a single industry category in a single commercial database did not yield a homogenous group of small food stores. Instead, the sample yielded a heterogeneous group of stores in terms of nutritious food options: some stores provided many nutritious food options and fresh fruits and vegetables, but most provided a limited variety of nutritious food items and produce. Store attributes (number of employees and sales volume) listed in the commercial database did not distinguish store type as well as the in-store survey and census data did. These findings reinforce those of previous studies that found significant discrepancies between store categorizations from secondary food retail databases and field observations (22-25) and suggest that database imprecision may introduce error or bias or both into public health and epidemiological research.

Commercial databases may not identify food stores in more deprived neighborhoods as accurately as they do in less deprived neighborhoods (25). This was not the case in our study. However, convenience stores (limited availability of nutritious foods) tended to be in more densely populated census tracts, and grocery stores (better availability of nutritious food) tended to be in tracts that had a higher percentage of whites. Convenience and specialty stores were found in tracts that had a higher average percentage of Asians. Powell et al also found differences in agreement on census tract race/ethnicity between field observations and proprietary database information for grocery stores in Chicago (24), corroborating evidence that discrepancies in measures of community nutrition environments do not vary randomly among all neighborhoods. Store visits may be necessary to obtain a more accurate understanding of the availability of nutritious food.

Our results show discrepancies between a commercial database and surveyed characterizations of store types across neighborhoods, thereby complicating efforts to quantify the availability of nutritious food in large areas by using commercial databases. Improving the availability of nutritious food items and fresh foods at small grocery, convenience, and specialty food stores is a promising approach for improving community nutrition environments in underserved communities (26). The national Healthy Food Financing Initiative allocated more than $400 million in 2011 to fund local, state, and regional collaborations that expand access to nutritious foods (27). Now that funding is available to support community nutrition environments, it is essential to identify accurately high-need areas that should be prioritized for intervention.

Our study had several limitations. The survey assessed the availability of nutritious food in each store but did not evaluate price or accessibility, such as proximity to public transportation, which could affect the ability of some people to access nutritious foods. We did not compare the availability of nutritious foods with energy-dense and snack foods, which are associated with body mass index (28) and fruit and vegetable intake (29), nor did we examine the proximity of each store to other food stores. This study used data that are not temporally consistent. Socioeconomic and demographic data were from the 2000 US Census, commercial data were from 2008, and surveys were conducted in 2009. The 1-year lag between the collection of data obtained from the database and the administration of the surveys may have contributed to our inability to locate 15% of the stores selected from the database, but other field validation studies of food stores have found similar rates of database overcounts (22,23). Our study results may not be generalizable to other areas. Each county in this study has a higher median household income than that of California and the United States.

Our study had several strengths. It is the first to compare data from in-store surveys of nutritious food availability at small food stores with data from a commercial database and data on socioeconomic and demographic characteristics. It demonstrates the use of a multidimensional approach to evaluate variability in community nutrition environments (30) by considering both the location and context of food stores and the food products offered.

The variables in a commonly used commercial database do not accurately correspond to the variables public health and epidemiology researchers are interested in, namely indicators of the availability of nutritious and fresh food. Industry classification for small food stores varies. Although conducting in-store surveys requires more time and resources than collecting information from a database, surveys may be necessary to assess accurately the food environment and identify where improved availability of nutritious food is most needed.

## Appendix. Northern CA Retail Food Environment Store Survey.

This survey is available for download in Microsoft Word format.
