# Supplementary material for: Small Food Stores and Availability of Nutritious Foods: A Comparison of Database and In-Store Measures, Northern California, 2009
Source: Prev Chronic Dis. 2012 Jul 12;9:E127. doi: 10.5888/pcd9.120023 (PMC3468308; doi:10.5888/pcd9.120023)
Supplement: Supplementary file 1 [file 12_0023_01.doc]

**Northern CA Retail Food Environment Store Survey**

**Adapted from the CX^3^ Food Availability and Marketing Survey created by the California Department of Public Health**

| **A. STORE INFORMATION**  **Date & Time Visited:________________**  **GPS Waypoint: ___________________**  **Surveyor Name(s):_______________________**  Disposition: **1** Completed  (circle one) **2** Partial  **3** Denied / No data  **4** Denied / Exterior only  **5** Store not found  **6**  Store closed  **7** Store not visited  **8**  Inaccessible |
| --- |

**1)** **Store Name**

**a.** Provided (CONAME/NETS/county): ______________________________

**b.** Actual (on storefront): ________________________________________

**2)** **Store Address** (#, street, city, zip)

**a.** provided: (NETS/county/Google): ____________________________________

**__________________________________________________________**

**b.** actual (on storefront): ________________________________________

**3)** **Store Location Description**

**a.** Streets (i.e. corner of X St. and Y St or on X St in between Y St and Z St):

______________________**______________________________________**

**b.** Side of street (circle one or more): North East South West

| **B. STORE EXTERIOR** |
| --- |

|  | **Circle One** |
| --- | --- |
| **4)** Are there any produce bins on the sidewalk in front of the store? | **1 - Yes 0 - No** |
| **5)** Are other products displayed on the sidewalk in front of the store or inside the  store *next to* the window so they are clearly visible from the outside? | **1 - Yes 0 - No** |
| **a.** Please check all that apply:  __ soda __ water __ other. (specify) __________________ | **🡸 If Yes** |
| **6)** Are there vending machines on the sidewalk in front of the store? | **1 - Yes 0 - No** |
| **a.** Please check all that apply:  __ soda __ water __ other. (specify) ____________________ | **🡸 If Yes** |
| **7)** Is there advertising (banners, posters, temporary signs, etc.) on the storefront? | **1 - Yes 0 - No** |
| **a.** Describe: | **🡸 If Yes** |
| **8)** Are there any images of **healthy** food (e.g. tomato, apple) and/or  beverages (e.g. milk), painted/displayed on doors or windows of the storefront? | **1 - Yes 0 - No** |
| **9)** Are there any images of **un-healthy** food (e.g. hamburger, hot dog) and/or  beverages (e.g., soda, shake) painted/displayed on doors or windows of the storefront? | **1 - Yes 0 - No** |
| **10)** Are there permanent bars/chains on the windows or doors? (do not include sliding or rolling chains or bars) | **1 - Yes 0 - No** |
| **11)** If the store sells alcohol, is more than 1/3 of the total window area covered by  any type of advertising? | **1 - Yes 0 - No 9 - N/A** (no alcohol) |

| C. STORE INTERIOR |
| --- |

**12)** Number of cash registers throughout store (staffed and unstaffed, for grocery items only)

**13)** Approximate area of store, in paces (food shelves/aisles area) : _____x_______

**14)** Circle the number that best describes overall availability of fresh produce and meats inside and outside the store.

|  | **None** | **Limited**  **(1-3 types)** | **Moderate variety**  **(4-6 types)** | **Wide variety**  **(7 or more types)** |
| --- | --- | --- | --- | --- |
| **a. Fresh fruit** | **1** | **2** | **3** | **4** |
| **b. Fresh vegetables** | **1** | **2** | **3** | **4** |
| **c. Raw meat/seafood** | **1** | **2** | **3** | **4** |

**15)** Circle the number that best describes the overall quality of the **fresh fruit**.

| **0** | **1** | **2** | **3** | **4** |
| --- | --- | --- | --- | --- |
| None sold | All or most of fruit is of poor quality (brown, bruised, overripe, wilted) | Mixed quality; more poor than good | Mixed quality; more good than poor | All or most of fruit is of good quality (very fresh, no soft spots, excellent color) |

**16)** Circle the number that best describes the overall quality of the **fresh vegetables**.

| **0** | **1** | **2** | **3** | **4** |
| --- | --- | --- | --- | --- |
| None sold | All or most of vegetable is of poor quality (brown, bruised, overripe, wilted) | Mixed quality; more poor than good | Mixed quality; more good than poor | All or most of vegetable is of good quality (very fresh, no soft spots, excellent color) |

**17)** Record if the following items are available in the store.

| **Food Item** | **Circle one** |
| --- | --- |
| a. Milk – (skim, low fat, or reduced fat), plain white (not flavored) | **1 - Yes 0 - No** |
| b. Soy beverage, plain, with no added sugar or sweeteners | **1 - Yes 0 - No** |
| c. Cheese | **1 - Yes 0 - No** |
| d. Eggs | **1 - Yes 0 - No** |
| e. Tofu, plain | **1 - Yes 0 - No** |
| f. Whole wheat bread | **1 - Yes 0 - No** |
| g. Brown rice | **1 - Yes 0 - No** |
| h. High fiber cereal (≥ 3 grams fiber, ≤ 12 grams sugar per serving) | **1 - Yes 0 - No** |
| i. Oatmeal (plain) | **1 - Yes 0 - No** |
| j. Tortillas, soft corn or whole wheat (no lard) | **1 - Yes 0 - No** |
| k. Beans, dried or canned with no added fats, sugar, or sweetener | **1 - Yes 0 - No** |
| l. Tuna (light), salmon, or sardines canned in water | **1 - Yes 0 - No** |
| m. Any canned fruit packed in 100% fruit juice | **1 - Yes 0 - No** |
| n. Any canned vegetable with no added fats, sugar, or sweetener | **1 - Yes 0 - No** |
| o. Any frozen fruit with no added fats, sugar, or sweetener | **1 - Yes 0 - No** |
| p. Any frozen vegetables with no added fats, sugar, or sweetener | **1 - Yes 0 - No** |
| q. Baby food, jarred, single fruit, single vegetable, or single meat | **1 - Yes 0 - No** |

**18) Please mark which of these most accurately describes the store:**

*_____* ***Large grocery store*** (not part of a large chain) - a large store that sells food and other items, including canned and frozen foods, fresh fruits and vegetables, and fresh (raw) and prepared meats, fish, and poultry. It may be part of a small regional chain of fewer than 5 stores or may be independent. (This type of store also has twenty or more employees and at least 4 cash registers).

*_____* ***Small Market*** - usually an independent store that may sell food including canned and frozen foods, fresh fruits and vegetables, and fresh (raw) and prepared meats, fish, and poultry as well as convenience items and alcohol. This type of store has fewer than 20 employees and three or less cash registers.

*_____* ***Convenience*** - a store that sells convenience items only, including bread, milk, soda, snacks and may sell alcohol and gasoline. These stores do not sell fresh (raw) meat. These stores also are known as food marts.

*_____* ***Other*** – Liquor Store, bakery, donut shop, meat or fish markets (predominantly selling fresh/raw meats), or other specialty stores.

***Please specify and describe: _______________________________________________________***
